# The Role of Lipid Metabolism in Influenza A Virus Infection

**DOI:** 10.3390/pathogens10030303

**Published:** 2021-03-05

**Authors:** Yong Zhou, Juan Pu, Yuping Wu

**Affiliations:** 1 Key Laboratory of Animal Epidemiology, Ministry of Agriculture, College of Veterinary Medicine, China Agricultural University, Beijing 100193, China; zhouyong@cau.edu.cn (Y.Z.); pujuan@cau.edu.cn (J.P.); 2 College of Life Science and Basic Medicine/Center for Biotechnology Research, Xinxiang University, Xinxiang 453003, China

**Keywords:** influenza A virus (IAV), host cell, lipid metabolism, replication

## Abstract

Influenza A virus (IAV) is an important zoonotic pathogen that can cause disease in animals such as poultry and pigs, and it can cause infection and even death in humans, posing a serious threat to public health. IAV is an enveloped virus that relies on host cell metabolic systems, especially lipid metabolism systems, to complete its life cycle in host cells. On the other side, host cells regulate their metabolic processes to prevent IAV replication and maintain their normal physiological functions. This review summarizes the roles of fatty acid, cholesterol, phospholipid and glycolipid metabolism in IAV infection, proposes future research challenges, and looks forward to the prospective application of lipid metabolism modification to limit IAV infection, which will provide new directions for the development of anti-influenza drugs.

## 1. Introduction

Influenza viruses are single-stranded, negative-sense RNA viruses that can be further classified into four types (A, B, C and D) based on the antigenic differences in the nucleoprotein (NP) and matrix protein (M). Among these types, influenza A virus (IAV) has the widest host range and is the most harmful. The IAV genome comprises eight segments encoding basic polymerase 2 (PB2), basic polymerase 1 (PB1), acidic polymerase (PA), hemagglutinin (HA), NP, neuraminidase (NA), M, and the nonstructural protein (NS) and can encode at least 10 essential and 7 accessory proteins [1,2,3,4,5]. A total of 18 different HA (H1–H18) and 11 different NA (N1–N11) subtypes of IAV have been identified in avian and bat species [6,7,8]. Therefore, IAVs are prone to antigenic mutations and subtype changes, leading to increased difficulty in prevention and control. Currently, H1N1 IAV and H3N2 IAV are the seasonal IAV subtypes circulating worldwide (https://www.who.int/zh/news-room/fact-sheets/detail/influenza-(seasonal), access on 28 January 2021) and cause tens of thousands of deaths each year [9]. Recently, frequent incidents of human infection by various avian influenza viruses (AIVs) that have crossed the species barrier, such as H5Nx, H7N9, and H9N2, have occurred [10]. In addition, H1N1 derived from swine influenza virus (SIV) caused a global pandemic in 2009 [11], and a recently reported G4 genotype of H1N1 SIV also has the potential risk of causing a human pandemic [12]. Therefore, the study of IAV infection mechanisms and prevention and control approaches remains an extremely urgent task worldwide.

**Figure 1:**
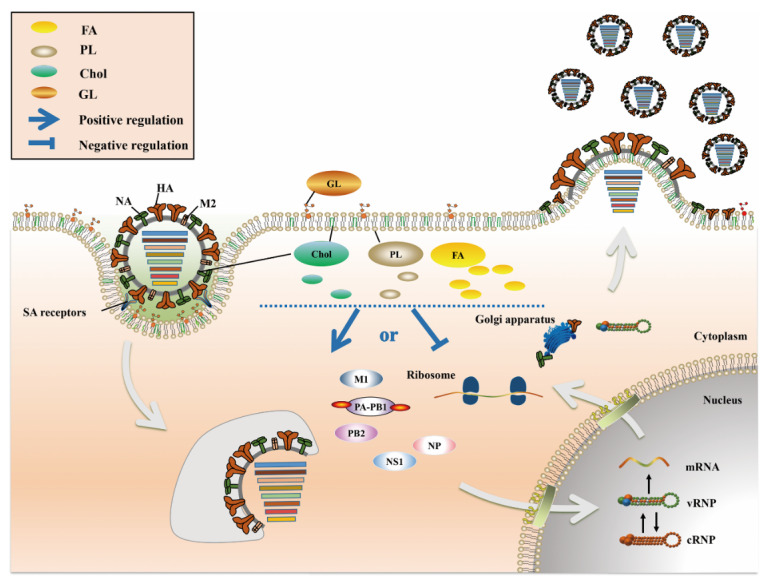
The role of lipid metabolism in IAV infection. FA (**yellow**), Chol (**green**), PL (**dark gray**) and GL (**brown**) are the four main lipid components present in cells, most of which are components of the biological membrane. Among these lipids, FA exist mainly in the cytoplasm, Chol and PL exist in the cell membrane and cytoplasm, and GL exist mainly in the extracellular space. After IAV infects host cells, a series of changes occur in lipid metabolism, which play a positive or negative role in multiple stages of the IAV life cycle.

IAV does not have its own metabolic system and must depend on the host cell metabolic system to complete its life cycle. IAV manipulates the cell to initiate metabolic reprogramming in order to meet all the biomolecular needs required for its replication, while a series of host metabolic pathways are also regulated to defend against IAV infection. Thus, the virus and the host exhibit a competitive relationship in terms of metabolism. IAV as an enveloped virus; its replication process is related more strongly to the host cell’s lipid metabolism than to other metabolisms. Lipids are widely distributed in living organisms, and not only are they an important cell structural component, but they also participate in various biological activities, such as cellular development, apoptosis, material transport and immune regulation. Cellular lipid metabolism is directly involved in almost all states of the viral life cycle (Figure 1), including the initial interaction of IAV with host cells, membrane fusion, nuclear import and export, and coordination of viral particle assembly and budding [13,14,15,16]. Moreover, lipid metabolism indirectly regulates IAV infection by affecting the immune system [17,18,19]. Numerous studies have shown that the characteristics of animal metabolites are greatly altered after infection with IAV [20,21] and have identified significant differences in the lipid composition of different pathogenic IAVs [22]. These results indicate that lipid metabolism not only plays a regulatory role in the process of IAV infection but also is closely related to the pathogenicity of IAV. In this review, we focus on the roles of fatty acid (FA), cholesterol (Chol), phospholipid (PL) and glycolipid (GL) metabolism in IAV infection. Understanding the relationship between lipid metabolism and IAV infection can help to further delineate the pathogenic mechanism of IAV and is highly important for the targeted development of vaccines and anti-influenza drugs.

## 2. Positive and Negative Regulation of FA Metabolism in IAV Infection

FA is closely related to the inflammatory and allergic responses of organisms and widely involved in the regulation of IAV infection. Studies have shown that compared with non-obese mice, obese mice will have more severe inflammatory reaction after IAV infection due to abnormal FA metabolism [23]. By analyzing the lipid metabolites of IAV-infected mice, Morita et al. also found that polyunsaturated fatty acids (PUFA) 12-hydroxyeicosatetraenoic acid (12-HETE), 15-hydroxyeicosatetraenoic acid (15-HETE), 17-hydroxy-4,7,10,13,15,19-docosahexaenoic acid (17-HDoHE) and omega-3 PUFA-derived lipid mediator protectin D1 (PD1) inhibited IAV infection after exogenous addition and reduced the lethality of IAV infection [21]. Further studies showed that PD1 specifically inhibited the nuclear export of IAV mRNA and suppressed viral replication [24]. Another in vitro study showed that lipoxygenin B4 (LXB4), derived from omega-6 PUFA arachidonic acid (AA), promoted the expression of B lymphocyte-induced maturation protein 1 (Blimp1) and X-box binding protein 1 (XBP1), the key transcription factors involved in plasma cell differentiation, thereby increasing the number of effector B cells and production of IgG, which promoted host resistance to IAV [25]. In addition, through the study on the basic metabolism of lung injury and multiple organ failure caused by human H7N9 virus infection, it was found that palmitic acid, a saturated fatty acid and erucic acid, omega-9 long chain monounsaturated fatty acid were negatively correlated with the severity of the disease, and could be used as key molecules for the prognosis of the disease [26]. In conclusion, FA such as PD1, LXB4, palmitic acid and erucic acid have negative regulatory effects on the infection process of IAV, which provides a scientific reference for the prevention and control of influenza and disease prognosis.

FA also have a positive regulatory effect on IAV infection that can exacerbate the severity of IAV infection. Studies have shown that mice fed long-chain PUFA such as eicosapentaenoic acid (EPA) and docosahexaenoic acid (DHA) have enhanced susceptibility to IAV, leading to increased morbidity and mortality [27], and further analysis revealed that EPA and DHA suppressed splenic natural killer (NK) cell activity, significantly reduced the number of CD8+ T cells in the lungs, and inhibited macrophage inflammatory protein-1α (MIP-1α), tumor necrosis factor-α (TNF-α) and interleukin-6 (IL-6) mRNA expression [28], thus severely suppressing the host immune response to IAV. Not coincidentally, prostaglandin E2 (PGE2) also helps IAV evade the host immune response, inhibiting the IFN-mediated innate response and adaptive immunity. After PGE2 binds to its receptors EP2 and EP4, it can activate intracellular phosphatidylinositol 3-kinase (PI3K)-Akt and protein kinase A (PKA) signaling pathways, thereby inhibiting IRF3-mediated type I IFN activation and antigen presentation as well as T cell activation, exacerbating the damage caused by IAV infection [29,30,31,32]. In addition, phospholipase D (PLD), a key enzyme in FA metabolism, can promote IAV replication by inhibiting the expression of myxovirus resistance gene A (MxA) [33]. In 2010, PLD was identified as a host factor required for IAV replication [34], and PLD has potential use as an anti-influenza target drug [35].

## 3. Chol Plays a Major Role in Fusion and Release during the IAV Life Cycle

Chol is an indispensable substance in cells, not only participating in the formation of cell membranes but also serving as a precursor for the synthesis of a variety of bioactive substances. Additionally, Chol is an important component of the IAV envelope, accounting for approximately 11–12% of its total mass [36], and is very important for IAV fusion and release [37]. After binding to the sialic acid receptor, the successful entry of IAV into the cell further depends on fusion of the viral envelope with the intracellular body membrane [38], and viral envelope Chol plays a key role in this process. Pretreatment of IAV particles with different concentrations of methyl-β-cyclodextrin (soluble Chol) resulted in a dose-dependent reduction in the cellular infectivity of IAV via solubilization of Chol, but this inhibition was limited to the viral fusion process [39,40]. In addition to the critical role in the entry stage as the viral envelope Chol, cell membrane Chol is essential to ensure effective budding of progeny virions; the infectivity and stability of progeny virions released from cells deficient in cell membrane Chol are severely impaired because progeny virions acquire their envelope from the host cell membrane during budding, and Chol plays a key role in maintaining the IAV envelope structure [41,42,43]. In vivo, Chol homeostasis is regulated by Annexin A6 (ANXA6) [44,45,46]. Overexpression of ANXA6 decreased Chol levels in the cell membrane, thereby inhibiting IAV replication and propagation, and when siRNA was used to reduce the expression of ANXA6, the titer of the progeny IAV virions was increased [47]. In addition, ANXA6 inhibits IAV budding by directly interacting with influenza matrix protein 2 (M2) [48]. The CRAC motif of the M2 protein is a potential binding motif for Chol, but whether ANXA6 competes with progeny virions for binding to the CRAC motif is unknown [49]. ANXA6 may be a target for anti-influenza drugs by regulating Chol [44].

It is worth mentioning that Chol distributed on the cell membrane forms lipid rafts together with sphingolipids [50,51] Host lipid raft is selected by IAV as a host attachment factor for multivalent binding, and play a critical role in assembly and budding of progeny virions. The polyvalent binding of IAV HA protein with sialic acid receptor will cause the aggregation of lipid rafts, which leads to the concentration and aggregation of tyrosine protein kinase (RTK) and activation, and then causes the response of PI3K/Akt and other related signaling pathways, and promotes the endocytosis of IAV [52,53]. IAV also uses the “lipid raft” region on the host’s cell membrane as the virus budding site. The two kinds of glycoproteins NA and HA of the virus will firstly gather in the lipid raft region, causing the membrane deformation and bending in the lipid raft region, and initiating the process of budding [54]. These indicate that the replication cycle of IAV depends on the lipid raft and is inseparable from Chol and sphingolipids.

## 4. PL Metabolism Is Involved in Multiple Stages of IAV Infection

PL includes mainly glycerophospholipids and sphingomyelin (SM), which are the main components of biological membranes. Glycerophospholipids can be further divided into many categories depending on the substitution groups, for example, phosphatidylcholine (PC), phosphatidylglycerol (PG), phosphatidylethanolamine (PE), phosphatidylserine (PS), phosphatidylinositol (PI) and cardiolipin (CL). As an enveloped virus, IAV needs to acquire an envelope from the host cell membrane during budding of progeny virions, which inevitably leads to changes in the PL content of the host cell. Alveolar type II (ATII) cells are the most important target cells of IAV; among the pulmonary surfactants synthesized by these cells, the PL content is as high as 35–50 mg/mL [55]. When infected by IAV, ATII cells undergo reprogramming of PL metabolism, and the cell surface levels of PC, PG and PE are reduced, while those of PS, PI and SM are increased [56].

Changes in glycerophospholipid levels affect IAV replication. On the one hand, glycosylation in the vicinity of cellular sialic acid receptors leads to blockade of virion binding to cellular receptors, but elevated PS levels can abolish this blockade and increase cellular susceptibility to enveloped viruses by up to 20-fold [57,58]. On the other hand, PI can exert anti-IAV effects by directly binding to IAV with high affinity, disrupting its adsorption to host cells and binding to sialic acid receptors [59]. In addition, PI can antagonize the activation of homologous ligands of Toll-like receptors (TLRs), central components of innate immunity, thereby inhibiting the transcription of proinflammatory genes, suppressing the cytokine storm induced by IAV infection and reducing damage to the organism [60,61]. This observation indicates that the reprogramming of PL metabolism is multifaceted and shows that during IAV infection, glycerophospholipids act not only as the body’s “guardian” against IAV but also as an “accomplice” of IAV.

**Figure 2:**
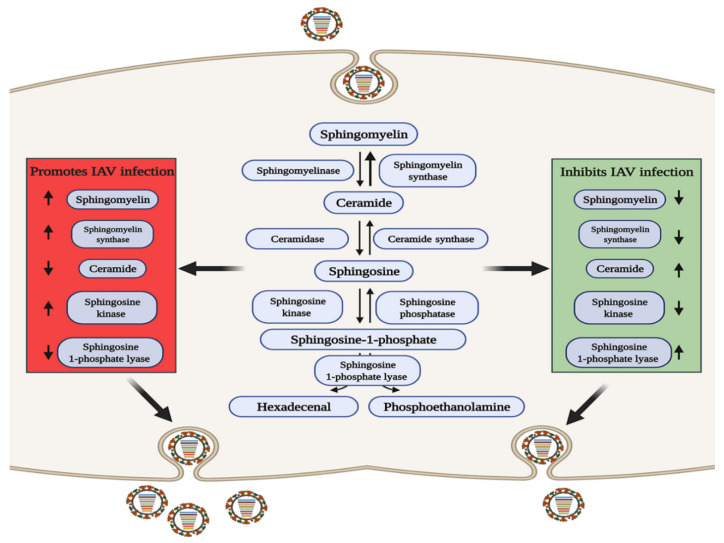
The role of sphingomyelin metabolism in IAV infection. In the red area, when the levels of sphingolipids or related metabolic enzymes are up-regulated or down regulated as shown by the arrow, IAV infection can be promoted through a variety of ways; conversely, in the green area, when the levels of sphingolipids or related metabolic enzymes are up-regulated or down regulated as shown by the arrow, IAV infection can be inhibited.

SM is of interest due to its important intracellular signaling and regulatory roles [61], and its three metabolites of ceramide (Cer), sphingosine (Sph), and sphingosine-1-phosphate (S1P) are biologically active signaling molecules [62]. SM and Cer play roles in promoting and inhibiting IAV infection, respectively, and some related metabolic regulatory enzymes involved in SM metabolism have been reported to have related effects (Figure 2). Increased levels of SM contribute to IAV infection, and sphingomyelin synthase (SGMS1), which catalyzes the production of SM from Cer, has a synergistic effect [63,64,65]. Audi et al. have proved that SM defects in the cell membrane and viral envelope inhibit viral infection by impairing virus adsorption and internalization [66], while in SGMS1-deficient cells infected with IAV, the transport of newly synthesized HA and NA proteins from the Golgi apparatus to the cell surface is inhibited, severely reducing the production of progeny virions [67]. In contrast, elevating the levels of Cer can inhibit IAV replication, and Cer can enhances the activation of dendritic cells (DCs), which enhances both CD8+ and CD4+ T cell responses to viral infection [68].

S1P is a terminal active metabolite of SM metabolism, and requires binding to different S1P receptor (S1PR1-S1PR5) to function. When S1PR1 is activated with CYM-5442, it can inhibit intercellular cell adhesion molecule-1 (ICAM1) production in a β-arrestin2-dependent manner, thereby reducing lung injury mediated by the strong immune response triggered by IAV infection, but it cannot reduce the viral titer [69,70,71], indicating that S1PR1 cannot reduce viral replication but can reduce pathological damage. Sphingosine kinase 1 (SPHK1) is the key enzyme that catalyzes the generation of S1P from Sph [72,73]. In infected cells, SPHK1 was shown to promote viral RNA synthesis by activating NF-κB and to maintain CRM1/RanBP3-mediated nuclear export of the viral ribonucleoprotein (vRNP) complex by activating ERK and AKT [74,75,76,77,78]. S1P lyase (SPL), which induces S1P degradation, has also been shown to participate in the host anti-IAV response. SPL induces rapid activation of ERK and STAT1 and enhances the activation of IKKε, thereby promoting the host immune response [79]. The above studies indicate that metabolites and enzymes in the SM metabolic pathway are widely involved in the regulation of IAV infection.

## 5. An Appropriate GL Concentration Is Important for IAV Infection

GLs are a class of amphiphilic lipid compounds containing glycosyl ligands that are widespread in living organisms, and glycosphingolipids (GSLs) are a main component of the GL family with multiple cellular roles. Some previous studies concluded that IAV must bind to cell surface GSLs to successfully infect cells [80,81], but other studies demonstrated that GSL is not required for IAV infection but acts as an adsorption stabilizer only during the entry stage of the IAV life cycle [82,83,84]. Glucosylceramide (GlcCer) is the main component of GSLs and can be converted into glucose and Cer under the catalytic activity of glucocerebrosidase (GBA) [85], while glucosylceramide synthase (GCS) catalyzes the synthesis of GlcCer from Cer. After using CRISPR-Cas9 to knock out GBA, the level of GlcCer was increased by approximately three- to four-fold, delaying the transport of IAV to late endosomes and inhibiting IAV infection [86]. In addition, when CRISPR/Cas9 was used to knock out the GCS gene, the level of GlcCer was reduced, and the infectivity of IAV was weakened [87]. The above findings suggest that either excessive or insufficient levels of GlcCer inhibit IAV replication, suggesting that an appropriate concentration of GlcCer is extremely important for maintaining optimal IAV infection ability without dose dependency.

## 6. Conclusions and Perspective

Recently, outbreaks of zoonotic diseases worldwide have shown a rapid upward trend. IAV is a zoonotic virus; once it becomes able to effectively infect and spread in human population, it may cause serious public health incidents. Currently, prevention and control of seasonal IAV rely mainly on vaccines, but due to the variable antigenicity of IAV, vaccine candidate strains need to be reevaluated and recommended by the World Health Organization (WHO) annually, which greatly increases the socioeconomic burden. Regarding drug therapy, the current selection of drugs available for treatment of the frequently mutating IAV is limited and includes mainly M2 ion channel inhibitors and NA inhibitors. Both amantadine and rimantadine target the M2 protein and are specific for IAV [88]. Oseltamivir and zanamivir, which target the NA protein, are active against IAV and influenza B virus (IBV) [89]; however, due to frequent mutations in the IAV genome, many IAV strains are already resistant [90], and the development of new anti-influenza drugs is thus urgently needed. Lipid metabolism is involved in multiple stages of IAV replication (Figure 1, Table 1) and can constitute a direction for future research on anti-influenza drugs. For example, the modulator of lipid raft structures composed mainly of Chol and SM is expected to become the target of new anti-influenza drugs [91,92,93,94].

The role of lipid metabolism in influenza virus infection is a new research direction, but most current research is phenotypic research. There are many research challenges we are facing, including two major issues. We want to know (a) which mechanism triggers changes in lipid metabolism during influenza infection and (b) how host lipid metabolism defends viral infection and recovers its normal level. More specifically, lipid metabolism is known to have an important balance including deficiency or overaccumulation of lipids; however, the current studies mainly focus on the effect of single specific lipid metabolite in IAV infection, and it is necessary to further understand the role of the balance between different lipid metabolites in viral infection. Moreover, changes in lipid metabolites level may not only inhibit or promote IAV infections but also cause metabolic disorders in the body. Therefore, it is necessary to systematically evaluate the pathogenic effect of IAV infection due to metabolic disorders and how the body responds and restores lipid metabolite balance to reduce the pathogenic effect. In addition, the specific mechanism by which many lipid metabolites exert their effects during IAV infection is currently unclear, and changes in lipid metabolites level rely mainly on the regulation of related metabolic enzymes; thus, the role of lipid metabolites in IAV infection, whether dominated by an enzyme itself or the lipid metabolites regulated by the enzyme, needs further clarification. Although many problems remain to be explored and solved, with the development and improvement of lipidomic techniques, the combined use of multiomics, and innovations in science and technology, the study of the mechanism by which lipid metabolism impacts IAV infection has become simpler and faster, allowing possible screening of lipid targets of anti-influenza drugs in the future.

## Figures and Tables

**Table 1:** The roles of lipid metabolism in IAV infection.

Type of Metabolism	Name of Component	Role in IAV Infection	Reference
FA	12-HETE, 15-HETE, 17-HDoHE	Inhibits IAV infection in vitro	[21]
	PD1	Inhibits nuclear export of IAV mRNA	[21,24]
	LXB4	Promotes the production of effector B cells and IgG	[25]
	EPA, DHA	Inhibits innate and adaptive immunity	[28]
	PGE2	Inhibits IFN-mediated innate immunity and impairs adaptive immunity	[32]
	PLD	Promotes host innate immune evasion by IAV	[33]
Chol	Viral envelope Chol	Promotes the fusion of IAV particles	[39]
	Cell membrane Chol	Improves the infectivity and stability of progeny virions	[41]
PL	PS	Promotes the susceptibility of cells to IAV infection	[57]
	PI	Binds to IAV with high affinity and inhibits inflammation induced by IAV infection	[60]
	SM	Promotes the fusion and infectivity of progeny virions	[61,66]
	Cer	Promotes the adaptive immune response after viral infection	[63]
	SGMS1	Promotes the release of progeny virions	[67]
	SPHK1	Promotes vRNA synthesis and vRNP nuclear export	[74,77,78]
	SPL	Enhances the immune response to IAV	[79]
GL	GlcCer	Maintains the optimal state of IAV infection	[86]

## Data Availability

Data is contained within the article.
